# Using Social Media for Clinical Research: Recommendations and Examples From the Brown-Lifespan Center for Digital Health

**DOI:** 10.2196/35804

**Published:** 2022-06-13

**Authors:** Elizabeth M Goldberg, Rochelle K Rosen, Don S Dizon, Kirsten J Langdon, Natalie M Davoodi, Tyler B Wray, Nicole R Nugent, Shira I Dunsiger, Megan L Ranney

**Affiliations:** 1 Department of Emergency Medicine Warren Alpert Medical School of Brown University Providence, RI United States; 2 Department of Health Services, Policy & Practice Brown University School of Public Health Providence, RI United States; 3 Brown-Lifespan Center for Digital Health Providence, RI United States; 4 Department of Behavioral and Social Sciences School of Public Health Brown University Providence, RI United States; 5 Department of Psychiatry and Human Behavior Warren Alpert Medical School of Brown University Providence, RI United States; 6 Department of Medical Oncology Warren Alpert Medical School of Brown University Providence, RI United States; 7 Lifespan Cancer Institute Rhode Island Hospital Providence, RI United States; 8 Department of Psychiatry Rhode Island Hospital Providence, RI United States; 9 Department of Emergency Medicine Providence, RI United States; 10 Center for Alcohol and Addiction Studies, Department of Behavioral and Social Sciences School of Public Health Brown University Providence, RI United States

**Keywords:** social media, Twitter, Facebook, clinical research, privacy, institutional review board, regulations, regulation, guideline, big data

## Abstract

Social media integration into research has increased, and 92% of American social media participants state they would share their data with researchers. Yet, the potential of these data to transform health outcomes has not been fully realized, and the way clinical research is performed has been held back. The use of these technologies in research is dependent on the investigators’ awareness of their potential and their ability to innovate within regulatory and institutional guidelines. The Brown-Lifespan Center for Digital Health has launched an initiative to address these challenges and provide a helpful framework to expand social media use in clinical research.

## Introduction

Social media includes technologies that allow multidirectional communication via web-based networks (Facebook), microblogs (Twitter), video sharing sites (YouTube), blogs, and other forums [1]. A 2021 Pew Research Center survey found that 72% of American adults use some form of social media, with that figure surpassing 80% among those under 50 years of age [2]. As social media use increases, its integration into and relevance for clinical research has also increased. These web-based channels offer a low- or no-cost venue for recruitment [3-6], more ready venues for volunteer engagement [7], and greater generalizability owing to the potential of web-based tools to access diverse or marginalized communities [6,8].

Beyond these aspects, social media also offers tremendous opportunities for clinical researchers. First, it provides the opportunity to increase knowledge about clinical research in a way that encourages the public to learn and discuss issues. For instance, during the COVID-19 pandemic, the National Science Foundation funded COVID Info Commons [9], a “convergence accelerator” that promotes federally funded research on COVID-19 on its Twitter account, provides the public with a search engine to find National Science Foundation–funded COVID research, holds monthly seminars over Zoom, accessible to the public, on research in progress, and posts recorded seminars to YouTube with Spanish and American Sign Language interpretation. Second, social media also allows for the delivery of interventions in an innovative way [8] and is a potential source of real-world evidence that can be accessed to generate new hypotheses or identify unmet needs in various clinical communities [10,11].

Despite the potential applications that can be used, social media research still faces significant barriers to its effective use. Principal among them is the lack of uniformity in how research proposals are reviewed at a local and national level and the lack of guidance available to researchers seeking to explore social media; this in turn may result in the unintended consequence of discouraging new and established researchers from incorporating social media into their own work.

The Brown-Lifespan Center for Digital Health (CDH) is a hub where researchers, clinicians, administrators, entrepreneurs, and business representatives from Brown University and its affiliated hospital partners collaboratively design, test, and deploy digital solutions to the society’s most pressing health challenges. In this paper, we review the issues facing investigators and institutions related to research using social media technologies. We propose a roadmap for researchers, agencies, and institutions to integrate social media as a tool for completing clinical research studies. We focus on elements of social media use investigators should be cognizant of, issues institutional review boards (IRBs) should address, and suggest institutional procedures to facilitate safe and responsible social media use for clinical research.

## Social Media and the Clinical Researcher: Concerns and Considerations

**Figure 1:**
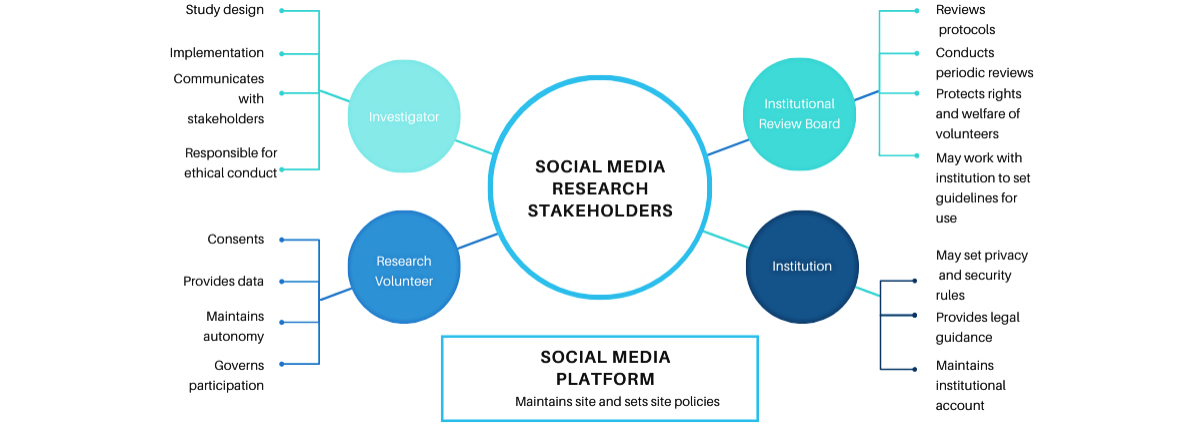
Social media research stakeholders.

While the potential role of social media in research has been established, issues have been raised by investigators, including ethics, privacy, consent, and confidentiality concerns for participants [12]. Additionally, whether and how communities on social media represent real-world or offline communities is a concern [13], especially as older and underresourced individuals may lack access to broadband or familiarity with social media channels; this inequality is often termed the “digital divide” [14]. Moreover, new social media platforms are constantly emerging, introducing dynamically changing impacts to participants and researchers, while also reshaping use patterns of more established tools. The stakeholders in social media research and their key roles are illustrated in Figure 1.

## Institutional Perspectives on Social Media Research: Concerns and Considerations

The use of social media for the purposes of human subjects research remains an area of concern for many institutions. The major reason this remains the case is that social media is not designed for ethical, human subject–approved research. Rather, it is designed, by intent, for public use. Further, social media platforms often include data agreements between the platform’s creators and all users that allow third parties to data mine in order to influence people using these platforms (eg, by targeted advertising or streamlined content). As a result, the intention for social media platforms is diametrically opposed to the most basic tenets of clinical research, including but not limited to the importance of deidentification and human subject protection. These concerns were succinctly brought forth in a recent paper by Vallury et al [15] in detailing their experience assessing public attitudes related to abortion in Australia. The authors describe how the lead researcher of a study on abortion stigma experienced “a barrage of harassment on and beyond social media” when her web-based research went “viral.” The lessons learned include the need for a supportive and coordinated institutional response to plan for and manage web-based and offline mental and physical health and safety risks. They recommend the development of training, guidelines, and policies to address the practical and ethical aspects of using social media for research.

Social media research requires an understanding of the following: the ethics of using web-based data as research data; the responsibilities of the researcher to participants both during and following the study; ensuring diversity and equity in who can access the study; and the risks and consequences to the researcher and the institution (particularly if the subject matter reflects politically or socially controversial topics). Ultimately, while the approach to social media research must be based on traditional understandings of good clinical practice and protections (for participants and for researchers), social media reflects an ever-changing environment that institutions must be prepared to recognize and effectively respond to.

## The CDH Proposal on Social Media Research Applications

As social media research proliferates, research practices in digital health and social media must similarly be regularly reviewed and updated so that they evolve as well. In short, best practices and local research guidelines need to be established and regularly updated to facilitate the protection of research study volunteers and the investigators involved.

In Table 1, we listed several critical questions that researchers should address during the *design* of a study that uses social media and provided suggestions as to how each can be approached. At the earliest stages, it is incumbent on research teams to establish norms for their social media research, including how to safeguard identifiable information, ensure privacy of both the study team and research participants, and maintain confidentiality of research documents. These plans should be provided as written documentation to the local IRB. Table 2 includes examples of how CDH-affiliated faculty used social media for research, including references.

**Table 1:** Questions for investigators to address during study planning.

Category	Issue	Critical questions	Suggested approaches
Approach	Recruitment	Will participants be recruited via traditional means (in research facilities, over the phone, or by flyers), by social media, or both? Will other strategies, such as crowdsourced or gig economy social media recruitment be used? What social media platform (eg, LinkedIn, Twitter, Instagram, Facebook, or Discord) will be used? Rationale? How will the investigator approach sampling? What social media account (eg, related to a research lab, an institution, or an investigator) will the research team use? Provide rationale. Will the participants be compensated?	Provide data on the demographics of the participants as these may vary depending on the social media network employed. Share how the participants will be routed from social media sites to Health Insurance Portability and Accountability Act–compliant data collection software sites to obtain further information. Obtain letters of agreement from social media account participants to collaborate (eg, institutional or influencer). Include social media community members in research design and implementation whenever feasible and appropriate.
Research team	Expertise	Who on your team has expertise in social media use?	Team members should have experience in social media research. If not available, ensure collaborators are involved who do.
Research plan	Dissemination	Will data sets collected over social media be shared? With whom? How? How will participants be informed of study progress and results?	Unless specifically approved otherwise, only share deidentified data. Informing participants of the study results is the responsibility of the research team.
Human subjects protection	Privacy and confidentiality	How will personal identifiers including social media account names be protected by the research team? What data will be obtained from social media? Will account analytics, such as on Twitter or videos, be used? What consent process will be used prior to data acquisition? Will teams verify the identities of social media participants? How? How will teams deidentify the accounts? Will the research team engage with participants via social media?	Be aware of the platform’s privacy and confidentiality policy [12]. Clarify what data are available publicly versus what data are available only with consent. If electronic consent will be used, describe the consent process and how participant comprehension is verified, and provide strategies to verify that the participant meets the eligibility criteria of the study. Clarify and assess understanding of protocols for social media posting (eg, participant-posted photos and video will be part of the research record), including who can create or add content and who will be able to see or use it. Define provisions to reduce risk of breach of confidentiality.
Human subjects protection	Security	How will information be collected and stored? How will the team ensure that third parties will not have access to information about the participant’s interests or affiliations?	Describe the process for the collection of public versus private data, and whether third-party services will be used to facilitate data collection. Specify that third parties will not have access to answers to investigator-posted surveys or screening instruments [4]. Be aware and describe relevant institutional policies on social media use.
Human subjects protection	Risks	How will disclosures of self-harm, trolling or other harmful comments, and other human subject concerns be monitored and identified? What is the crisis mitigation plan if disclosures are identified?	Describe strategies for mitigating and addressing risks to participants (eg, as described by Nicholas et al [10] in “risk detection”), including frequency of monitoring, anonymity of subjects, and crisis mitigation plans. Disclose to participants that you will not be monitoring their responses in real time, and provide them with a document or create a blanket post that lists resources for immediate help.
Human subjects protection	Recruitment	If material will be posted on social media for purposes of recruitment: Where will the ads be posted? Will the ads be targeted to certain demographics? How? How will ambient privacy be maintained?	Provide examples of the kinds of ads or communication that may be used in the study. In order to harness the social media networks’ full potential to build community, investigators may need to be agile, and it is not feasible to submit verbatim advertisements and communication to the institutional review board.
Human subjects protection	Equity and diversity	What strategies will be used to ensure recruitment includes women, minorities, and other underrepresented communities?	Describe plans to ensure equitable access to recruitment and estimate likelihood of recruitment of demographic subgroups. Consider the fact that recruitment techniques that enroll web-based participants looking for paid work (eg, through MTurk or Craigslist) may result in more demographically diverse participants than those that use a recruiting ad (eg, Facebook) [16].
Protection of the study team	Risks	What are the foreseeable risks to the study team in the conduct of this research? What is the plan to mitigate these risks?	Restrict social media communication to handles specific to the study, not to any one individual on the research team. Avoid using personal social media handles to communicate research-related activities.

**Table 2:** Examples from the Center for Digital Health faculty illustrating how social media can be used for clinical research.

Use category	Study topic and authors	Notes
Recruitment	Telehealth in Older Adults, Goldberg et al [17]	Physicians were recruited into qualitative interviews through advertisements posted on Twitter, Facebook physician groups, and specialty society and physician listservs
Identified individuals for an intervention	A Cyberbullying Media-Based Prevention Intervention for Adolescents on Instagram: Pilot Randomized Controlled Trial, Kutok et al [18]	Recruited a national sample of adolescents with a history of past-year cybervictimization through Instagram for a randomized control trial delivered via an app-based program.
Idea generation, iterative improvement of app based on participant feedback, and dissemination	MyCovidRisk—a free app to help individuals assess their risk of being infected with COVID-19, Goldberg et al [19]	A collaboration was formed between 2 investigators after a Twitter conversation about the need of an app that assists the public with assessing COVID-19 risk. Then, the investigators crowdsourced opinions on risk categories and what was considered an “acceptable” risk by the public on Twitter. The investigators shared a beta version of the app on Twitter and modified the design and content based on public feedback. Finally, information about how to access the app was advertised on Twitter and other social media channels.
Performed a needs assessment	The Needs of Women Treated for Ovarian Cancer: Results From a #gyncsm Twitter Chat, Thomas et al [20]	Investigators obtained IRB^a^ approval to conduct a tweet chat asking women about survivorship from ovarian cancer. Questions were asked surrounding needs after cancer treatment, and the responses were analyzed quantitatively and qualitatively.
Used Twitter to obtain data from users in a specific location and analyzed the results qualitatively	#PuertoRicoSeLevanta: A Closer Look at the Language Used on the First-Year Anniversary of Hurricane Maria, Rodríguez-Guzmán et al [21]	In order to examine psychological processes 1 year after Hurricane Maria and understand the differences in reactions depending on location, the research team collected tweets using hashtags associated with Hurricane Maria and geomapping. They used Linguistic Inquiry and Word Count software (LIWC2015, Pennebaker) to conduct a quantitative linguistic analysis of the sample of tweets.
Created a novel data set using crowdsourcing	Crowdsourcing from Scratch: A Pragmatic Experiment in Data Collection by Novice Requesters, Papoutsaki et al [22]	Used crowdsourcing techniques and Amazon Mechanical Turk to create a data set of all Computer Science faculty in the 50 top Computer Science graduate programs. This project yielded guidelines that novice requesters can use who are new to using crowdsourcing for data collection and extraction from the web.
Obtained insights on human affect	Sochiatrist: Signals of Affect in Messaging Data, Massachi et al [23]	Extracted social media data and deidentified them to understand how messages can serve as a proxy for changes in a person’s affect.

^a^IRB: institutional review board.

## Practical Guidance for Institutional Review Boards

We recommend that IRBs develop policies surrounding the appropriate and safe use of social media in clinical research. Sharing these guidelines with researchers who plan to use social media in their studies will help ensure consistency and can be useful for investigators and IRBs alike to improve efficiency and reduce the need for revisions. Gelinas et al [24] created an IRB checklist for evaluating social media recruitment proposals that can be a valuable resource for this purpose. Below, we summarize major considerations related to recruitment, benefits, risks, and informed consent.

## Recruitment

**Figure 2:**
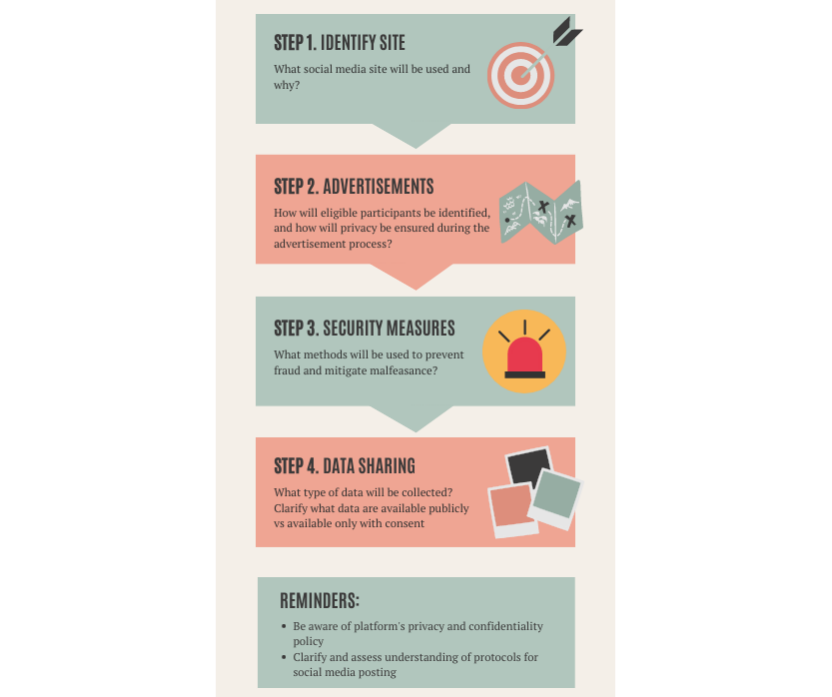
Steps in the institutional review board (IRB) process pertaining to social media–based recruitment methods.

The IRB application should specify which social media sites will be used and why, whether advertisements of the study will be used, and how targeted recruitment will be conducted, if applicable. For instance, recruitment advertisements posted on social media may draw global participation. Because of this, investigators should specify how they will ensure only eligible participants in the preferred geographic region will be recruited. Typically, this can be addressed by the inclusion of appropriate eligibility criteria as a part of a screening survey. The steps in the IRB process pertaining to social media–based recruitment methods are illustrated in Figure 2.

When using social media to recruit, researchers should put safeguards in place. Investigators should be aware that they may receive survey replies from fictitious accounts or be the target of harassment or other trolling behaviors seeking to discredit the study. Several methods exist to address these concerns [15], including the following: (1) offering compensation for users to verify that they are indeed who they are and delaying payment until completed; (2) regular and routine monitoring of advertisements and posts related to the study; (3) understanding the policies governing privacy, harassment, and reporting on the channels being used; (4) adopting mechanisms to moderate posting on public forums related to the research; (5) if surveys are used, users should take advantage of security measures to prevent fraud and mitigate malfeasance (eg, they can use Completely Automated Public Turing Test question types to prevent bots from submitting survey responses); in addition, proactively monitoring times to completion can be useful, as in our experience, completion rates are typically very fast for fictitious accounts; and (6) it is important to monitor the referring link to determine if links are being reshared for fraudulent purposes. Several surveys also allow investigators to prevent multiple submissions from one device by using cookies.

Researchers should acknowledge and disclose terms of sites or apps before advertising recruitment on them to avoid third-party data sharing. For example, research participants who engage with study advertisements could—depending on the type of advertisement and social media site—allow third-party sites to collect information about their interests or affiliations unknowingly [25]. Participants are often unaware of the terms of the apps and sites they use regularly. Investigators should be knowledgeable of these terms before they advertise their studies on these sites. In one study of men who have sex with men [25], participants had few concerns about data being shared anonymously with researchers but expressed more concerns with data being sold to third-party partners. However, research participants evidenced substantial variability in privacy concerns and comfort with sharing different types of data, suggesting a need to gain consent for data sharing for specific types of data.

The investigator should also specify how potential participants’ privacy will be protected during the advertising process. For example, a study recruiting people with a history of substance use disorder should be careful not to inadvertently violate participants’ privacy by advertising imagery or language that labels a potential participant as a person with an addiction. Research participants may not be forthcoming or truthful with their answers if they are particularly concerned about evading law enforcement [26], which may raise data quality concerns that should be addressed by the investigator. Investigators should educate their study participants about the limits of their confidentiality as needed.

## Benefits and Risks of Social Media Use for Research

The benefits of social media use for research include (1) ease of recruitment; (2) increased engagement by social media participants; (3) rapid sharing of information in a way that is intuitive for participant; (4) and building of web-based communities; moreover (5) several studies have shown that participants feel web-based participation in research is more private than in-person participation.

The risks of using social media for research include the following: (1) third-party use of data such as tracking participants’ clicks on advertisements; (2) breach of confidentiality through intentional or unintentional sharing of data by participants or the study team; and (3) exposure to malicious content; however, if the investigator is only using social media to recruit, there is no additional exposure to malicious content outside of what is seen from scrolling through your feed.

## Informed Consent

Researchers using social media for their studies may choose to offer research volunteer electronic informed consent (e-consent) if there is no waiver of consent. e-Consent refers to the use of electronic systems and processes to inform research participants of information related to the study and obtain and document their consent. In guidance prepared jointly by the Department of Health and Human Services and the Food and Drug Administration [27] for investigators, sponsors, and IRBs, the following recommendations were made for e-consent: (1) e-consent should be designed to convey information about the study to the research volunteer or their legally authorized representative in language that is understandable; (2) e-consent should allow navigation forward or backward so that participants can review information, and hyperlinks can be used to view further detail; (3) participants should have the option to use paper-based consent or be assisted by study personnel if they cannot use the e-consent technology; (4) study personnel should verify identity through a state-issued identification, the use of personal questions, biometric methods, or visual methods. Verification using these techniques may not be necessary in social behavioral minimal risk research studies; (5) opportunities to ask questions and consider participation are necessary; questions can be answered in person, over the phone, or by video conferencing, but should be answered prior to consent; (6) investigators should assess understanding of the study (eg, by including questions that test understanding or through other methods to gauge individuals’ comprehension of all elements of the consent; (7) participants should obtain a copy of the informed consent; and (8) IRBs should review the usability of the e-consent material to ensure they are easy to navigate and should review any optional questions or other methods used to gauge comprehension of key study elements.

An important aspect of consent relates to vulnerable populations, including but not limited to children and prisoners. For these participants, it will be important to request that researchers provide information to prevent coercion and a means to affirm consent, respectively. Finally, provisions for re-consent are necessary if the child comes of age during the study or if cognition improves or worsens during a longitudinal study in older adults [28].

## Confidentiality, Security, and Privacy

Investigators should describe how privacy protections are put in place for participants. For instance, are apps “sandboxed” so that apps on the same device cannot obtain data that the participant enters into the research app? If data are being collected, where will they be stored and who maintains access? For instance, most volunteers understand that if they post on social media sites publicly, their information will be discoverable by any user of the social media site. However, volunteers may not know that if they use more private ways to communicate with the research team, their information can still be retained by the platform. Twitter, for instance, allows participants to use “Direct Messages” to have nonpublic conversations on the platform. While these direct messages are not public per se, Twitter still stores and processes the communication and information shared in these messages [29]. For instance, links shared in direct messages are scanned for malicious content. Further, Twitter will not use the content of your message; however, information about whom you communicated with and when will be examined to better understand platform usage in an effort to generate more relevant content. Volunteers should also be aware that even if they delete their copy of the direct message, recipients (in this case, the research team) will retain their own copy, which they can duplicate, store, or reshare.

Other relevant questions include the following: Will participants have a right to view or edit their data? Moreover, how do you protect the privacy of parties who have not consented? For instance, for studies on Facebook, if you are an investigator and you are observing a research volunteer’s feed, you may see comments on the feed by their unconsented friends. It is important that researchers have a plan to include or exclude data from people not consented. These details require careful thought and consideration prior to initiating recruitment via social media platforms to ensure the protection of human subjects.

Investigators should also consider that third parties may develop novel ways to broach the security of platforms and exploit the identifiers of account holders. For instance, the administrator of a Facebook group, consisting of individuals who tested positive for breast-cancer mutations, discovered a Chrome extension that allowed marketers to scrape the membership lists of closed Facebook groups [30]. Facebook had previously added tools to make the membership lists of closed groups private, and they were unaware of this Chrome extension until the group administrator worked with a security researcher to submit the information to Facebook. Facebook then sent a cease-and-desist letter to the Chrome extension.

## Statistical Analysis

IRBs should be aware of unique uses and analytic techniques for social network analysis. Social network analysis often involves large samples and can have substantial computational requirements. For instance, in a study aiming to discover emergent web-based communities of cannabis participants for public health surveillance [8], investigators performed social network analysis by first finding the actors of interest, 6 cannabis dispensaries in Oakland, and then discovering accounts that follow these 6 accounts and their followers. Then, participant information was collected from these accounts such as friend counts, follower counts, and account creation date. The total number of accounts collected by these means included 2.2 million participants. Then, researchers used stochastic block modeling to infer network structure with the purpose of uncovering hidden populations of cannabis consumers. After manual coding, communities of illicit, recreational, and medical cannabis participants were identified. This analysis helped researchers examine a research question and illicit use patterns that would be challenging and costly to discover without social media analyses. However, these methods are computationally complex and require expertise in big data (analysis and data management) beyond what many investigators may need for traditional clinical research studies. Investigators need to be skilled in these advanced statistical techniques, such as stochastic block modeling and high-dimensional multilevel models, as well as qualitative content analysis, in order to identify spam and fraudulent accounts and ensure the validity of their findings. It is important to note that this level of work often requires significant server space and power to run the analyses; this availability may vary depending on institutional resources.

## Institutional Procedures to Facilitate Safe and Effective Social Media Use

Institutions may opt to publish social media guides when used for research to help investigators follow institutional privacy and security recommendations and to help them follow best practices in social media use. For instance, the Harvard Clinical and Translational Science Center publishes a guide, “The Use of Social Media in Recruitment to Research: A Guide for Investigators and IRBs,” that summarizes their laws and regulations, including Health Insurance Portability and Accountability Act, advises on recruitment techniques that follow their social media guidelines, and assists investigators in designing procedures that respect ethical norms [31]. The University of South Florida provides specific parameters for their faculty and staff to guide the development of a social media presence [32]. A mixed methods study including interviews with 5 institutional offices and 15 subject-matter experts at the University of Florida suggests that a centrally managed social media account for communicating with participants and initiating advertising campaigns could be successful to facilitate participant enrollment in health and clinical research studies [33]. Some institutions list social media accounts that have pre-existing approval for research usage [34,35]. However, if an institutional account is not already approved, it is recommended that the social media or public relations team from the institution work with the IRB and Human Subjects Protective Program to agree on guidelines for social media use in clinical research. Given that terms of agreement often include legal jargon, which may be confusing for investigators and research volunteers, it can be helpful to involve the institution’s legal team to help with interpreting terms of the chosen social media platforms.

## Conclusion

The right to privacy and data security is a fundamental aspect of clinical research that must be considered in the social media space. Researchers are expected to uphold the principles of trust and respect by approaching the aims and details of the study with transparency and refraining from collecting data about potential participants in ways unknown to the social media participant. Communication between the research team and research participants should be carried out in such a way to avoid breaches of confidentiality or exposing personal information in the public domain. Because communication may be frequent and cannot always be completely scripted on social media sites, it is beneficial for IRBs and institutions to agree to norms that allow the investigator to have flexibility to communicate with research participants in a manner that is consistent with the study aims and the IRB protocol. Finally, given the ever-changing terms of use and privacy policies on social media sites, it is critical for study teams to maintain awareness of such policies and develop plans to ensure ongoing compliance.

Further work is needed to (1) identify what unique safeguards may be necessary for individuals with special situations that make them more vulnerable to exploitation (eg, undocumented individuals, minors, and sex workers), (2) develop recruitment techniques and interventions tailored to special populations who are traditionally disadvantaged by the digital divide (eg, older individuals and rural persons), (3) suggest ways researchers can best recruit volunteers and access data from social media sites while being sensitive to the diverse privacy needs of volunteers (eg, different comfort levels with disclosure), (4) ensure all stakeholders understand the limitations of different platforms’ privacy policies, and (5) develop best techniques to disclose and increase the comprehension of yet unidentified vulnerabilities in platforms that can be exploited by third parties.

Social media can be a valuable tool for clinical research recruitment, retention, data collection, and dissemination. However, as an open and shareable entity, there is a possible dissonance between traditional research ethics and the public use of social media sites. Social media research stakeholders should be aware that our understanding of the ideal privacy policies and other safeguards for volunteers are still evolving and will likely never be static. Regulatory agencies, such as IRBs, and funding agencies should share clear guidelines for social media use in research to enhance innovation and ensure privacy and efficiency.

## Abbreviations

CDH: Center for Digital Health
e-consent: electronic informed consent
IRB: institutional review board
